# A Study of Mechanics in Brittle–Ductile Cutting Mode Transition

**DOI:** 10.3390/mi9020049

**Published:** 2018-01-29

**Authors:** Gaobo Xiao, Mingjun Ren, Suet To

**Affiliations:** 1 School of Mechanical Engineering, Shanghai Jiao Tong University, Shanghai 200240, China; gaobo.xiao@sjtu.edu.cn; 2 Department of Industrial and System Engineering, The Hong Kong Polytechnic University, Hong Kong 999077, China; sandy.to@polyu.edu.hk

**Keywords:** ultra-precision machining, brittle–ductile cutting mode transition, mechanics analysis, critical undeformed chip thickness, silicon carbide

## Abstract

This paper presents an investigation of the mechanism of the brittle–ductile cutting mode transition from the perspective of the mechanics. A mechanistic model is proposed to analyze the relationship between undeformed chip thickness, deformation, and stress levels in the elastic stage of the periodic chip formation process, regarding whether brittle or ductile mode deformation is to follow the elastic stage. It is revealed that, the distance of tool advancement required to induce the same level of compressive stress decreases with undeformed chip thickness, and thereby the tensile stress below and behind the tool decreases with undeformed chip thickness. As a result, the tensile stress becomes lower than the critical tensile stress for brittle fracture when the undeformed chip thickness becomes sufficiently small, enabling the brittle–ductile cutting mode transition. The finite element method is employed to verify the analysis of the mechanics on a typical brittle material 6H silicon carbide, and confirmed that the distance of the tool advancement required to induce the same level of compressive stress becomes smaller when the undeformed chip thickness decreases, and consequently smaller tensile stress is induced below and behind the tool. The critical undeformed chip thicknesses for brittle–ductile cutting mode transition are estimated according to the proposed mechanics, and are verified by plunge cutting experiments in a few crystal directions. This study should contribute to better understanding of the mechanism of brittle–ductile cutting mode transition and the ultra-precision machining of brittle materials.

## 1. Introduction

Brittle–ductile cutting mode transition is an important phenomenon in the ultra-precision machining of brittle materials [1,2,3,4]. Almost all the brittle materials could be machined in the ductile mode when the undeformed chip thickness ($t_{undeformed}$) decreases to be sufficiently small, typically at submicron level [5,6,7]. The mechanism of brittle–ductile cutting mode transition has been a hot topic over the past two decades, and has been explained from several perspectives, including the energy of the material removal, the effects of the preexisting microdefects, and the effective rake angle at small undeformed chip thickness, etc.

The theory based on the energy of the material removal was proposed by Bifano et al. [2]. The core idea of this theory is that the energy of the brittle mode material removal is proportional to the second power of the machining scale while the energy of the plastic flow is proportional to the third power of the machining scale, which means that the ductile mode material removal would be energetically more favorable when the machining scale is small enough. This theory is widely adopted to guide the ultra-precision machining of brittle materials, and modelling approaches based on this theory have been proposed to predict the critical undeformed chip thickness [3,8,9].

The theories from the perspective of preexisting microdefects [10,11] assumed that there are many microdefects in engineering materials and thereby some defects would be contained in the stress field of the cutting zone. Since the size of the stress field would decrease with the machining scale, the number of defects included in the stress field would also decrease with the machining scale, which improves the fracture toughness and eliminates brittle fracture. Other studies [12,13,14,15] also considered that the stress intensity around the preexisting cracks or defects would decrease with the machining scale and thereby would become insufficient to cause propagations of the preexisting defects when the machining scale becomes very small.

The theories from the perspective of the effective rake angle [16,17,18,19,20,21,22,23] pointed out that the effective rake angle would be extremely negative when the undeformed chip thickness is at the same order of the cutting edge radius. The extremely negative rake angle would induce high compressive stresses in the cutting zone and prevent the initiation or propagation of cracks. The high hydrostatic pressure induced by the extremely negative rake angle is reported to cause phase transformations to metallic phases, and account for the ductile regime machining of several semiconductors [24,25,26,27]. The influence of cooling methods on the thickness and shape of chip has also received significant attention [28,29,30,31].

In addition, there are also some insights from the molecular dynamics (MD) simulations. Shimada et al. [11] concluded from their MD simulation of nanomachining of silicon that the energy of shock waves emitted from the cutting zone would increase with the machining scale and cause crack propagation under large machining scales. Cai et al. [32] conducted MD simulations of nanomachining of silicon and proposed that the transition to brittle mode cutting at larger cutting thickness was due to the decrease of compressive stress with the increase of cutting thickness. Xiao et al.’s MD simulations [33] of orthogonal cutting of 6H-SiC indicate that the tensile stress around the cutting zone increases with the undeformed chip thickness and accounts for the brittle fracture under larger undeformed chip thicknesses. 

The previous studies have found that compressive stress would induce plastic flow in the cutting zone when the machining scale is very small [16,17,18,19,20,21,22,23], and that tensile stress around the cutting zone would cause brittle fractures at larger machining scales [22,33,34,35]. However, the relationship between the compressive and tensile stresses, and how their roles shift with the change of undeformed chip thickness have not been explicitly understood. In this study, the mechanism of brittle–ductile cutting mode transition is analyzed from the perspective of the mechanics in the cutting zone. The proposed theory is examined by finite element method (FEM) and plunge cutting experiments on a typical brittle material 6H silicon carbide (SiC). This study is expected to contribute a new insight into the mechanism of brittle–ductile cutting mode transition and help to achieve a more comprehensive understanding on it.

## 2. Mechanics of Brittle–Ductile Cutting Mode Transition 

Ductile mode cutting is usually a periodic process in which each cycle consists of two stages, i.e., a stage dominated by elastic deformation and a stage dominated by plastic deformation [36]. In the elastic stage, the tool compresses the workpiece material elastically to increase the compressive/shear stresses until the critical compressive/shear stresses are reached. The plastic stage follows the elastic stage after the critical stresses are reached, and plastic flow would happen in this stage to enable the chip formation. The plastic flow would also lead to the release of the strain energy and consequently the decrease of the compressive/shear stresses, until the compressive/shear stresses could not sustain the plastic flow. The elastic stage would again occur and this periodic process would continue to form the cutting chips. This is also the reason why for almost all the materials the thickness of the cutting chip is not constant and exhibits periodic variations. For cutting of brittle materials, this periodic process has two possible forms depending on the cutting mode, i.e., periodic elastic–plastic stages for ductile mode cutting and periodic elastic–brittle stages for brittle mode cutting. In brittle mode cutting, a brittle fracture stage would follow the elastic stage after the critical tensile stress for brittle fracture is reached. In this study, the stress states in the elastic stage would be analyzed, in terms of how undeformed chip thickness determines whether a plastic stage or a brittle fracture stage is to follow the elastic stage.

For geometrical simplification, an orthogonal cutting model is adopted to describe the machining process. Since the undeformed chip thickness ($t_{undeformed}$) in ductile regime machining of brittle materials is usually at the submicron level, the behavior of single grits on the grinding wheel should resemble that of negatively raked cutting tools, and thereby could also be described by this model. Under the configurations of orthogonal cutting and plunge cutting, $t_{undeformed}$ is equivalent to cutting depth. However, for practical machining processes like grinding and turning, $t_{undeformed}$ has quite significant difference with cutting depth, and is determined by several parameters like cutting depth, tool nose radius or wheel diameter, feed rates, and workpiece rotational speed or wheel speed. Though an orthogonal cutting model was adopted in this study, the nomenclature of $t_{undeformed}$ was adopted to avoid confusion with cutting depth in practical grinding or turning processes.

**Figure 1:**
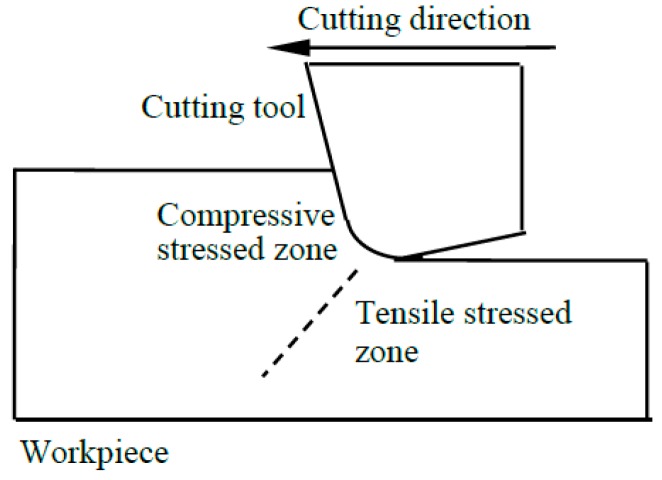
Schematic drawing of stress states in orthogonal cutting.

Figure 1 shows an orthogonal cutting model with a negatively raked cutting tool. The movement of the cutting tool would compress the workpiece material ahead of it, and at the same time stretch the workpiece material behind and below it. In other words, the advance of the cutting tool would induce compressive stresses in front of the tool [23,37,38], whereas tensile stresses would be induced behind and below the tool [34,35,39,40]. When the compressive stress exceeds the critical compressive stress $\sigma_{cc}$ of the workpiece material, plastic flow would occur and the material would be removed through ductile mode chip formation. It needs to be mentioned that the stress distribution in Figure 1 is a simplified illustration, whereas the actual stress distribution would be much more complicated. The stress distribution in a practical machining process would not only be influenced by the machining conditions, but also be affected by the crystal anisotropy [5,38,40,41,42,43,44]. Nevertheless, compression and tension would still exist in the zones before and behind the tool respectively. 

**Figure 2:**
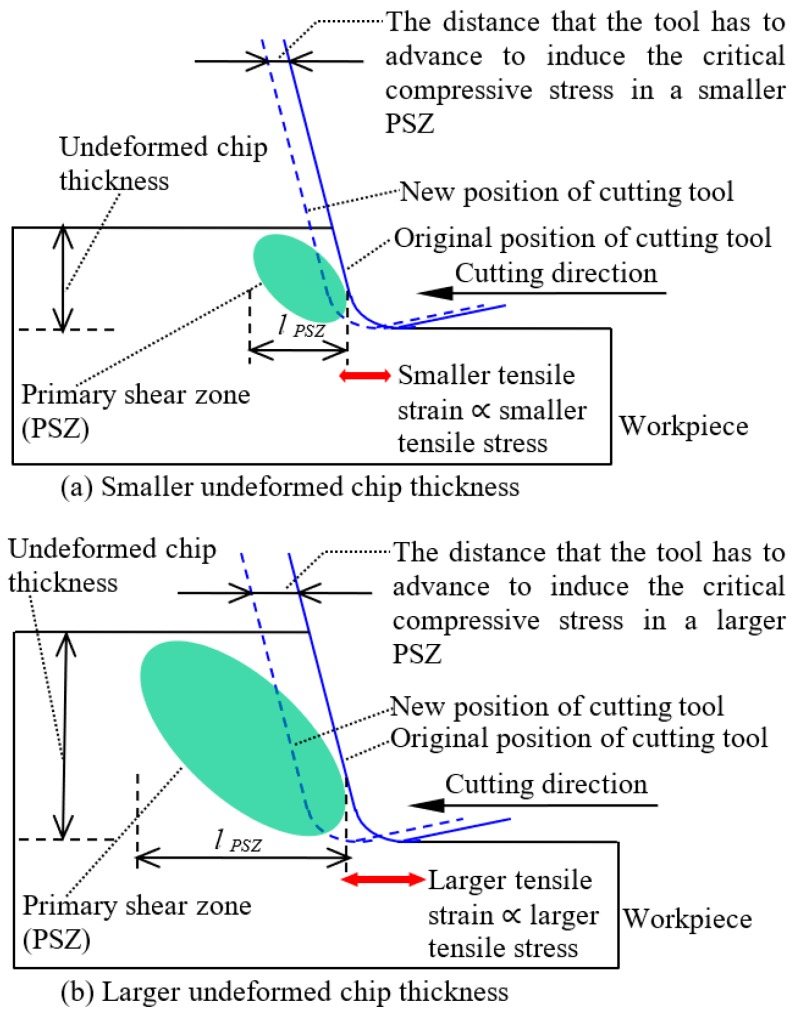
Illustration of how undeformed chip thickness affects the stress states.

Figure 2 shows how the undeformed chip thickness would influence the stress states in the cutting zone. In ductile regime machining, plastic flow occurs in the primary shear zone (PSZ) when the compressive stress around the PSZ exceeds the critical compressive stress $\sigma_{cc}$ for plastic flow [16,17,18,19,20,21,22,23]. The size of the PSZ ($l_{PSZ}$) would increase with the increase of undeformed chip thickness ($t_{undeformed}$), as illustrated by Figure 2, and their relationship could be expressed as

(1) $$l_{PSZ}\;\propto \;t_{undeformed}$$

Since similar compressive strains ($\varepsilon_{compressive}$) are required to induce similar levels of compressive stress around the PSZs under different undeformed chip thicknesses, a larger displacement of compression would be required to induce the critical compressive stress $\sigma_{cc}$ around a larger PSZ, which means that the tool has to advance for a larger distance ($d_{tool}$) to induce the critical compressive stress $\sigma_{cc}$ under a larger undeformed chip thickness. This could be expressed as

(2) $$d_{tool}\;\sim \;\varepsilon_{compressive}\cdot l_{PSZ}$$

A larger distance of tool advancement ($d_{tool}$) means a larger tensile strain ($\varepsilon_{tensile}$) in the workpiece material below and behind the tool, which thereby induces larger tensile stresses ($\sigma_{tensile}$) in these zones, since

(3) $$\sigma_{tensile}\;\propto \;\varepsilon_{tensile}\;\propto \;d_{tool}$$

Considering Equations (1)–(3), the tensile stress ($\sigma_{tensile}$) below and behind the tool would increase with the increase of undeformed chip thickness ($t_{undeformed}$), which could be expressed as

(4) $$\sigma_{tensile}\;\propto \;t_{undeformed}$$

When the tensile stress exceeds the critical tensile stress $\sigma_{ct}$ of the workpiece material, brittle fractures would be induced, forming subsurface cracks or leading to brittle mode material removal when the tensile stress becomes high enough to propagate the cracks [22,33,34,35]. It needs to be mentioned that Equation (4) is an approximate relationship since there are a few approximations in the derivation, e.g., the required compressive strain $\varepsilon_{compressive}$ is assumed to be constant for different undeformed chip thicknesses. Although the exact relationship between the tensile stress $\sigma_{tensile}$ and the undeformed chip thickness $t_{undefomed}$ would be affected by many factors, the basic relationship that $\sigma_{tensile}$ increases with $t_{undeformed}$ remains.

In summary of the mechanics of brittle–ductile cutting mode transition, the tool has to advance for a larger distance to induce the critical compressive stress in a larger PSZ under a larger undeformed chip thickness and thereby causes larger tension in the workpiece material below and behind the tool. As a result, the tensile stress below and behind the tool would increase with the undeformed chip thickness, and finally induces brittle mode material removal. Viewing these relationships from another perspective, the tensile stress around the cutting zone would reduce with the decrease of undeformed chip thickness, and thereby would become insufficient to induce brittle fractures when the undeformed chip thickness becomes small enough, enabling the brittle–ductile cutting mode transition.

It needs to be pointed out that the proposed mechanics does not contradict previous theories on the mechanism of brittle–ductile cutting mode transition, such as high pressure phase transformation (HPPT) [24,25,26,27] and the effects of preexisting defects [10,11,12,13,14,15]. For the theory of HPPT-induced ductile mode material removal, a certain level of compressive stress is required to induce the phase transformation and thereby the ductility, so it is consistent with the proposed mechanics in that both of them require a critical compressive stress to induce ductile deformation. As for preexisting defects in the material, they would influence the critical tensile stress for brittle fracture. Therefore, the effects of preexisting defects could also be considered in the proposed mechanics by taking into account their effects on the critical tensile stress. 

## 3. Examination of the Theory on 6H-SiC

In order to examine the mechanics proposed in the previous section, FEM models of orthogonal cutting of single crystal 6H-SiC were built to analyze the stress states under a series of undeformed chip thicknesses. Before the finite element analysis (FEA) could be performed, the critical tensile stress $\sigma_{ct}$ for brittle fracture and the critical compressive stress $\sigma_{cc}$ for plastic deformation of 6H-SiC in a few crystal orientations have to be determined. However, it is difficult to obtain these properties by experiments. Firstly, the current experimental techniques are usually indentation scale processes (usually around 10 μm) [45], while the scale of ductile regime machining is usually smaller than 1 μm. Secondly, it is difficult to measure the accurate tensile strength of brittle materials, as the results might be affected by preexisting flaws in the macro-scale samples, which would be quite rare in the micro-machining scales [11]. This is also the reason why existing data from macro-scale samples could not be adopted in this study. For these reasons, this study calculates the critical stresses by MD simulations. As for the elastic moduli of 6H-SiC, reliable data are already available from the literature [46] and are adopted for the FEM modelling.

### 3.1. Molecular Dynamics (MD) Simulation of Mechanical Properties

**Figure 3:**
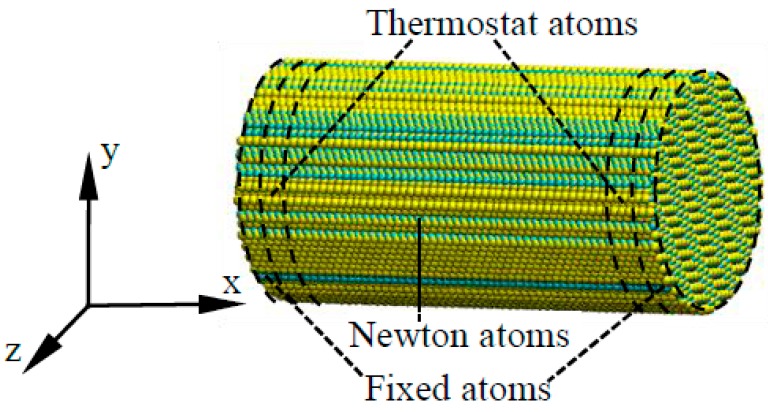
Molecular dynamics (MD) model for tension and compression tests.

MD simulations of uniaxial tension and compression tests were performed to calculate the critical tensile stress $\sigma_{ct}$ and critical compressive stress $\sigma_{cc}$ of 6H-SiC in a few crystal orientations. The MD model for uniaxial tension and compression tests of 6H-SiC is shown in Figure 3. The boundary conditions include two fixed boundaries and two thermostat regions in the two ends respectively, as shown in Figure 3. The temperature in the thermostat regions is kept constant at 300 K during the whole simulation to simulate the cooling effects of the environment on the specimen. The atoms in the center of the workpiece are Newton atoms, the motion of which followed Newton’s law of motion during the simulations. A limitation of the MD modelling is that it is conducted under much higher strain rate than that in the machining process, and the critical stresses might be influenced. However, the objective of the FEM modelling is to verify the relationship between undeformed chip thickness and stress states, and the exact values of critical stresses are less important here.

The interaction potential used in this study was the effective many body potential developed by Vashishta et al. [47], which has very good performance on reproducing the brittle fracture and high pressure phase transformation (HPPT) of SiC [47,48]. The MD simulations were performed using customized MD codes developed by the authors, which were based on the compute unified device architecture (CUDA) and utilized the arithmetic computation power of graphics processing units (GPU). The details of the MD codes were described in ref. [33]. The simulations were performed on a NVIDIA GeForce GTX 580 GPU (NVIDIA, Santa Clara, CA, USA). The integration time step was set as 1 fs in the simulations. The lattice constants of 6H-SiC, *a* and *c*, were set to be 3.073 Å and 15.08 Å respectively [49]. The MD simulations were conducted under the micro canonical (NVE) ensemble. The MD model was visualized by visual molecular dynamics (VMD) [50].

Each simulation was divided into two periods, a relaxing period and a loading period. During the relaxing period, the workpiece was relaxed for 10 ps in order to achieve a steady status of energy. During the loading period, the atoms in the two fixed boundaries moved in opposite directions at a constant speed to simulate the compression or tension process. The total strains for the compression and tension simulations were 0.4 and 0.2, which last for 40 ps and 20 ps respectively.

**Figure 4:**
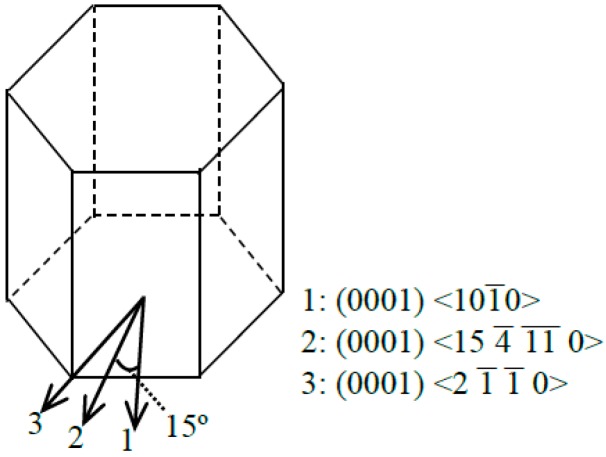
Crystal orientations for tension and compression.

Since 6H-SiC has sixfold symmetry on the (0001) basal plane; its mechanical properties will exhibit a periodic variation on the (0001) plane with a cycle period of 60°. Due to this reason, MD simulations of tension and compression tests were performed in three crystal orientations within 30°, which is half of a cycle period, as shown in Figure 4. The properties in symmetrical orientations could be obtained by applying crystal symmetry. The detailed simulation parameters are summarized in Table 1. 

**Figure 5:**
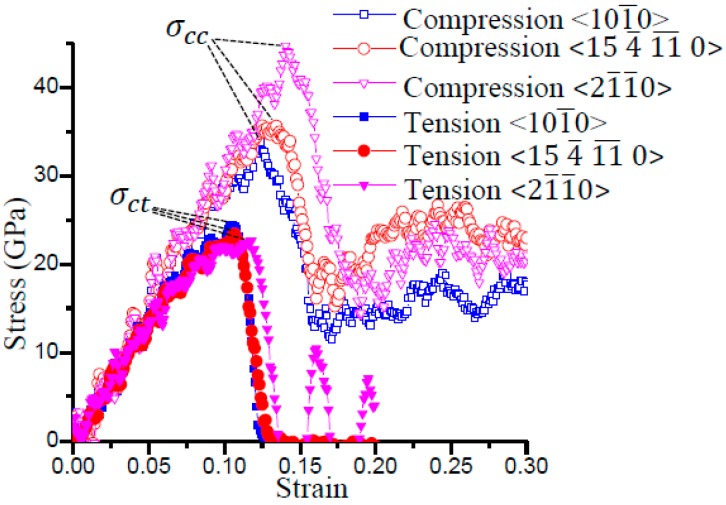
Stress-strain curves from MD simulations.

The stress–strain curves of 6H-SiC obtained by the MD simulations are shown in Figure 5. It can be seen that the critical compressive stress $\sigma_{cc}$ is generally higher than the critical tensile stress $\sigma_{ct}$, and that the variation of critical compressive stress $\sigma_{cc}$ with the crystallographic orientation is more significant than that of the critical tensile stress $\sigma_{ct}$. According to Figure 5, the values of the critical tensile stress $\sigma_{ct}$ and the critical compressive stress $\sigma_{cc}$ in the three orientations are summarized in Table 2.

### 3.2. Finite Element Method (FEM) Modelling of the Stress States

**Figure 6:**
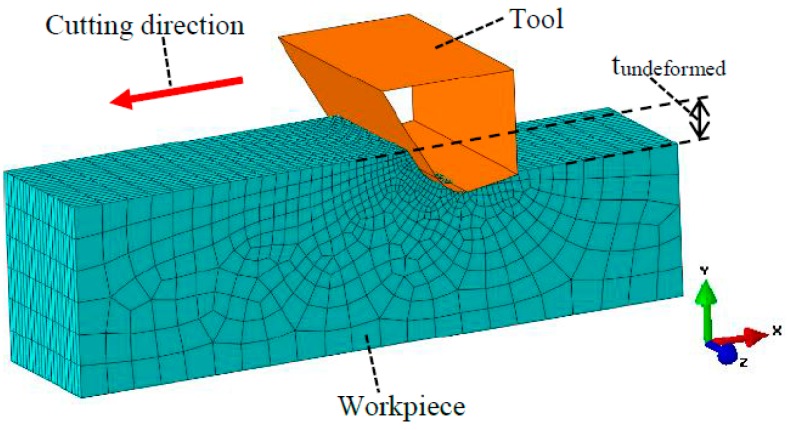
Elastic deformation finite element method (FEM) model of orthogonal cutting.

Only elastic deformation was considered in the FEM modelling, since the subsequent plastic deformation or brittle fracture is largely dependent on the stress states developed in the elastic stage. A series of FEM models with different undeformed chip thicknesses were built using Abaqus (ABAQUS Inc., Johnston, RI, USA), and the FEM model with the undeformed chip thickness of 50 nm is shown in Figure 6. The mesh type was C3D8R, with minimum mesh size of 5 nm. For each undeformed chip thickness, three sets of FEM modelling were performed with the mechanical properties in the three orientations denoted in Figure 4, respectively. The loading criterion for the FEM modelling was that, for each undeformed chip thickness and each cutting orientation, the tool would be moved forward at a constant speed of 3 mm/s until the maximum compressive stress $\sigma_{xx}$ reached $1.5\sigma_{cc}$. It was assumed that when the maximum compressive stress $\sigma_{xx}$ reached $1.5\sigma_{cc}$, the compressive stress in the cutting zone would be sufficient to induce ductile mode chip formation. Adopting $\sigma_{xx}$ as the criterion is a simplification of the real situation, since in a real machining process the ductile deformation would be determined by the overall stress states but not only $\sigma_{xx}$. As it is difficult to establish a comprehensive criterion considering all the stress components and other factors such as temperature and strain rates, a simplified criterion using $\sigma_{xx}$ would be appropriate since that the accurate values of stresses are not within the scope of this study. The reason why the criterion was set as $1.5\sigma_{cc}$ but not $\sigma_{cc}$ is that, when the maximum $\sigma_{xx}$ just reaches the $\sigma_{cc}$, only the $\sigma_{xx}$ in a very small area contacting the tool reaches $\sigma_{cc}$ and the $\sigma_{xx}$ in other areas would still be much lower than $\sigma_{cc}$. Since the stress states in a real machining process would be very complicated, it is difficult to find a perfect criterion that describes the real situation. The choosing of $1.5\sigma_{cc}$ as the criterion here is, in a sense, kind of trial and error. However, the core idea is that the compressive stress needs to reach a certain level so as to enable ductile mode chip formation. Since the major purpose of the FEM modelling was to qualitatively investigate the relationship between the undeformed chip thickness and the stress states, the determination of an accurate loading criterion would be less important here.

The FEM simulations were carried out using Abaqus. The elastic moduli of 6H-SiC were adopted from Adachi’s handbook on physical properties of semiconductors [46]. A friction coefficient of 0.1 was adopted to describe the friction between the tool and the workpiece. The effects of temperature were not considered in this study. The boundary conditions include fixed boundaries in the bottom and the two ends of the workpiece, and symmetrical boundaries along the z direction. The detailed simulation parameters are summarized in Table 3. 

**Figure 7:**
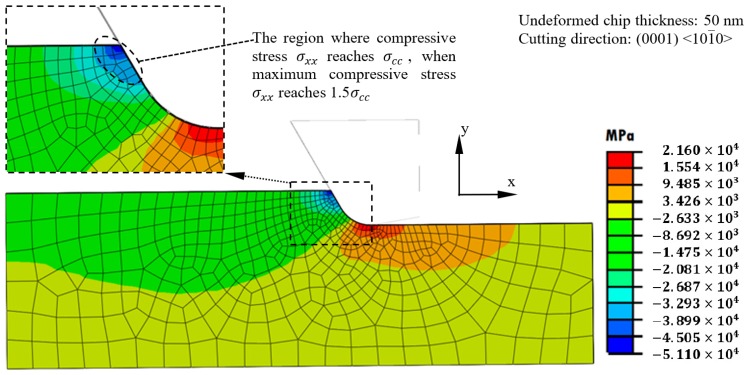
Distribution of $\sigma_{xx}$ in the cutting zone by FEM modelling.

Figure 7 shows the FEM-modelled distribution of stress $\sigma_{xx}$ in the cutting zone under the undeformed chip thickness of 50 nm and the cutting direction of (0001) <10$\overset{-}{1}$0>. It can be seen that compressive stress existed before the tool rake face, while tensile stress existed below and behind the cutting edge, which agreed with the illustration in Figure 1. The maximum compressive stress is 51.1 GPa, which corresponds to $1.5\sigma_{cc}$ in the orientation of (0001) <10$\overset{-}{1}$0>. The region where the compressive stress $\sigma_{xx}$ reaches $\sigma_{cc}$ is marked out in the enlarged view in the upper left corner of Figure 7. This region is still not very large as compared with the primary shear zone. However, since the cutting is a non-linear process, it is possible that the compressive stress in the other areas would become sufficient to induce ductile deformation after ductile deformation has happened in this small region under high compressive stress. Therefore, the loading criteria for all the simulations was set as that the tool move forward until the maximum compressive stress $\sigma_{xx}$ reached $1.5\sigma_{cc}$. 

**Figure 8:**
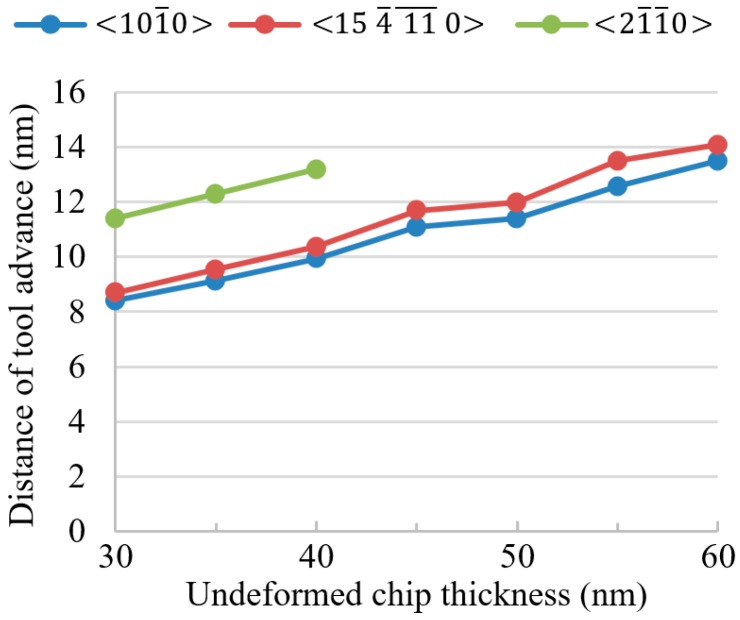
Distance of tool advancement to induce the required compressive stress.

Figure 8 shows the distances of tool advancement before the maximum compressive stress $\sigma_{xx}$ reached $1.5\sigma_{cc}$, under different values of undeformed chip thickness. It can be seen that for all the three cutting orientations, the distance of tool advancement required to induce the same level of compressive stress increased with the undeformed chip thickness. This agreed very well with the mechanics analysis in Section 2.

**Figure 9:**
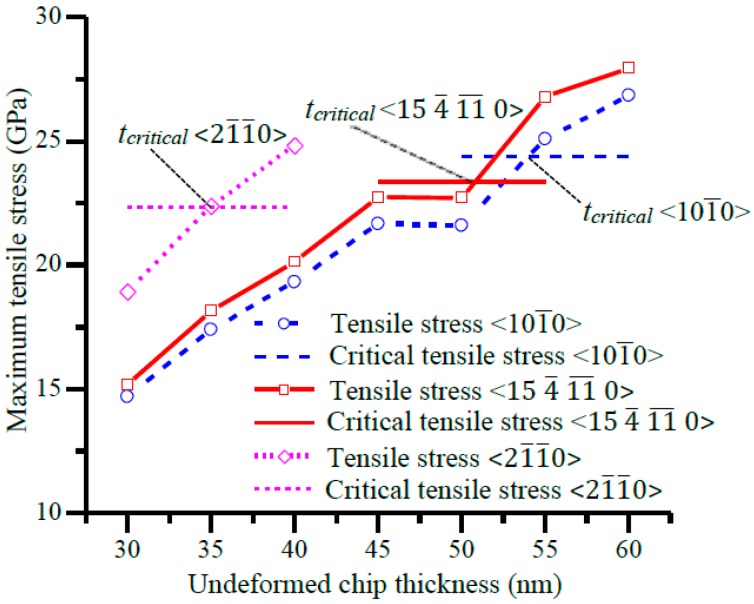
Relationship between tensile stress and undeformed chip thickness.

The maximum tensile stress $\sigma_{xx}$ in the workpiece when the maximum compressive stress $\sigma_{xx}$ reached $1.5\sigma_{cc}$ was assumed as the maximum tensile stress $\sigma_{xx}$ that would occur in the cutting process under a certain undeformed chip thickness and a certain cutting direction. The maximum tensile stresses $\sigma_{xx}$ under various undeformed chip thicknesses in the three cutting directions are shown in Figure 9. For each cutting direction, the horizontal line refers to the critical tensile stress $\sigma_{ct}$ for brittle fracture in that direction. It can be seen that, to reach a same magnitude of maximum compressive stress, the maximum tensile stress increases with the undeformed chip thickness. This agrees with the theoretical deductions in Section 2 that the magnitude of tensile stress in the cutting zone would increase with the undeformed chip thickness. 

Another point in Figure 9 is that, for each cutting direction, the maximum tensile stress $\sigma_{xx}$ is below the critical tensile stress $\sigma_{ct}$ when the undeformed chip thickness is very small, but would exceed the $\sigma_{ct}$ when the undeformed chip thickness becomes large enough. It could be expected that brittle fracture would occur when the tensile stress exceeds $\sigma_{ct}$, and if the brittle fracture propagates to remove the material, the cutting mode would transfer from the ductile mode to a brittle mode. Therefore, the intersection point between the increasing tensile stress $\sigma_{xx}$ and the critical tensile stress $\sigma_{ct}$ in a certain cutting direction could be approximately considered as the critical undeformed chip thickness $t_{critical}$ for brittle–ductile cutting mode transition. By linear interpolation, the undeformed chip thicknesses at the intersection points were calculated to be 54.0 nm, 50.8 nm and 34.9 nm for the <10$\overset{-}{1}$0>, <15 $\overset{-}{4}$ $\overset{-}{11}$ 0> and <2$\overset{-}{1}$$\overset{-}{1}$0> directions respectively. The critical undeformed chip thicknesses in symmetrical directions can be obtained by applying crystal symmetry, which would be discussed together with the experimental results in the next section.

It needs to be pointed out that assuming the intersection point of $\sigma_{xx}$ and $\sigma_{ct}$ in the cutting direction as the critical undeformed chip thickness is a simplification of the real situation. This is because the stress states around the cutting zone would be complicated in a practical cutting process and brittle fracture might be caused by tensile stresses in other directions instead of the cutting direction. This simplification might over-estimate the critical undeformed chip thickness, as the tensile stresses in other directions might have already caused brittle fracture before $\sigma_{xx}$ exceeds $\sigma_{ct}$ in the cutting direction. 

In summary, the FEM results show that, in order to reach a same magnitude of maximum compressive stress, the tensile stress would increase with the undeformed chip thickness, and would exceed the critical tensile stress for brittle fracture when the undeformed chip thickness increases to a certain level. This is consistent with the mechanics of brittle–ductile cutting mode transition proposed in Section 2.

### 3.3. Experimental Verification

**Figure 10:**
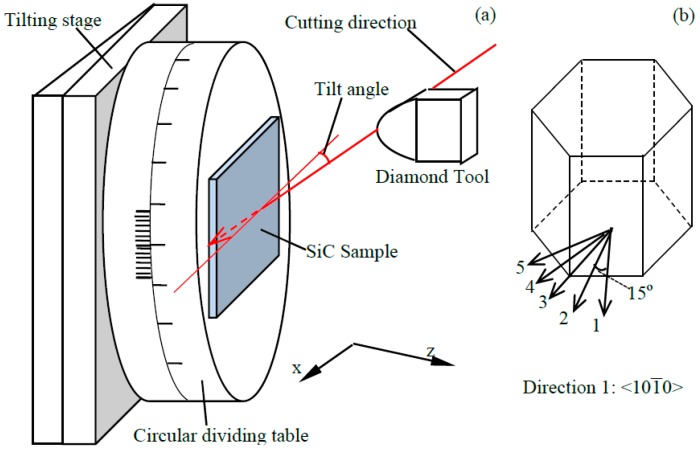
(**a**) Plunge cutting setup and (**b**) cutting directions.

Plunge cutting experiments were carried out on a Optoform 30 (AMETEK, Berwyn, PA, USA) ultra-precision turning machine to investigate the critical undeformed chip thickness of 6H-SiC in a few crystal directions. The illustration of the experimental setup is shown in Figure 10a. The tilting stage and the circular dividing table were fixed on the *z* slide of the Optoform 30 machine. The 6H-SiC sample was glued on the circular dividing table so that plunge cutting experiments could be taken out in different crystal directions. The tilting stage can tilt the sample by a small angle so that plunge cutting could be realized. The 6H-SiC sample was cleaved out from a two-inch single crystal 6H-SiC wafer from Xiamen Powerway Advanced Material Company (Xiamen, China). The original two-inch wafer had an orientation of (0001), and the thickness of the wafer was 410 µm. The primary flat of the wafer was in the <10$\overset{-}{1}$0> direction and served as the reference for the cutting directions. Since SiC has very high hardness and thereby it was unfeasible to conduct a facing operation before the plunge cutting experiments, the tilt angle was slightly different in different cutting directions. 

A single crystal diamond tool was adopted for the plunge cutting experiments. The cutting edge radius of commercial diamond tools are reported to be on the order of ~50 nm [51], and was not measured in this study. The nose radius of the diamond tool was 1.507 mm. The tool was fixed on the *x* slide of the Optoform 30 machine. During the cutting process, the *x*-slide feed at a constant speed towards the sample, while the *z* slide of the machine was fixed. A total of five cutting orientations were adopted in the experiments, as shown in Figure 10b. The detailed cutting parameters are summarized in Table 4. The cutting directions will be referred to as direction 1, 2, 3, 4 and 5 respectively in the following context, as denoted in Figure 10b. Since tool breakage is very easy to occur under high cutting speeds for cutting SiC, a cutting speed of 3 mm/s was adopted. This is reasonable since the purpose of the experiment is to support the mechanics analysis in Section 2 and FEM modelling in Section 3.2.

**Figure 11:**
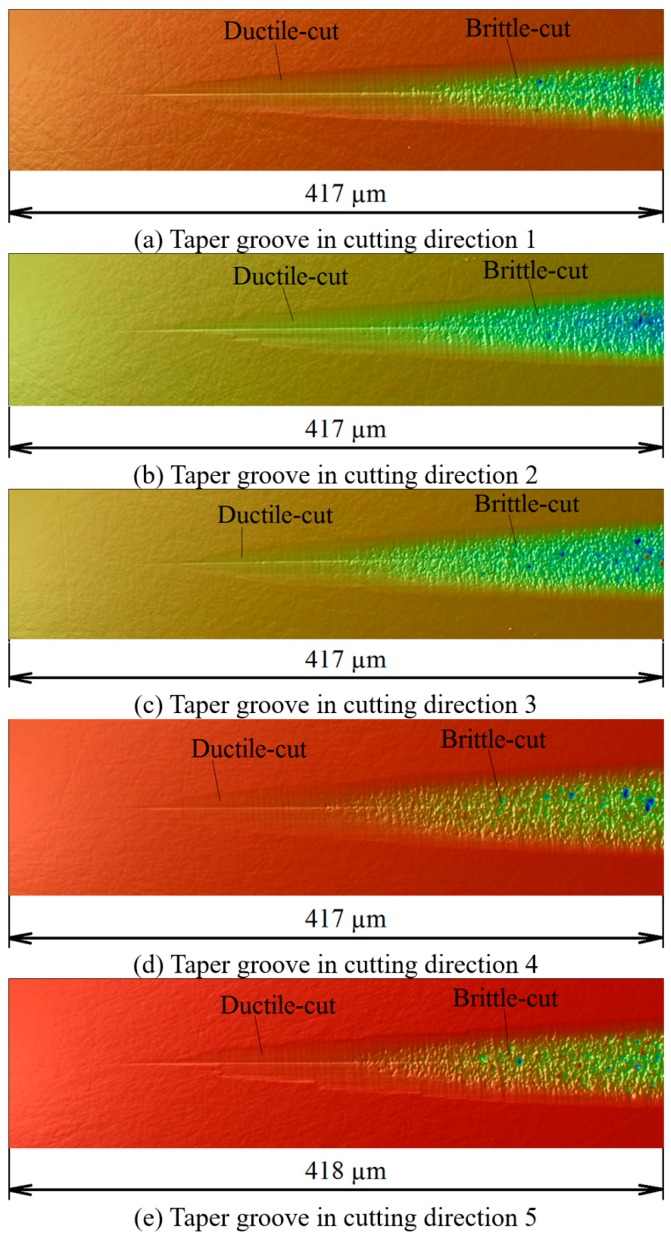
3D morphologies of the taper grooves.

The plunge cutting experiments generated five taper grooves on the 6H-SiC sample. The taper grooves were measured by a white light interferometer [52], and their surface morphologies were shown in Figure 11. For each groove, the morphologies in the ductile-cut region and the brittle-cut region are significantly different from each other. The surface in the ductile-cut region is almost as smooth as the original surface, while the surface in the brittle-cut region is very coarse and rugged. 

**Figure 12:**
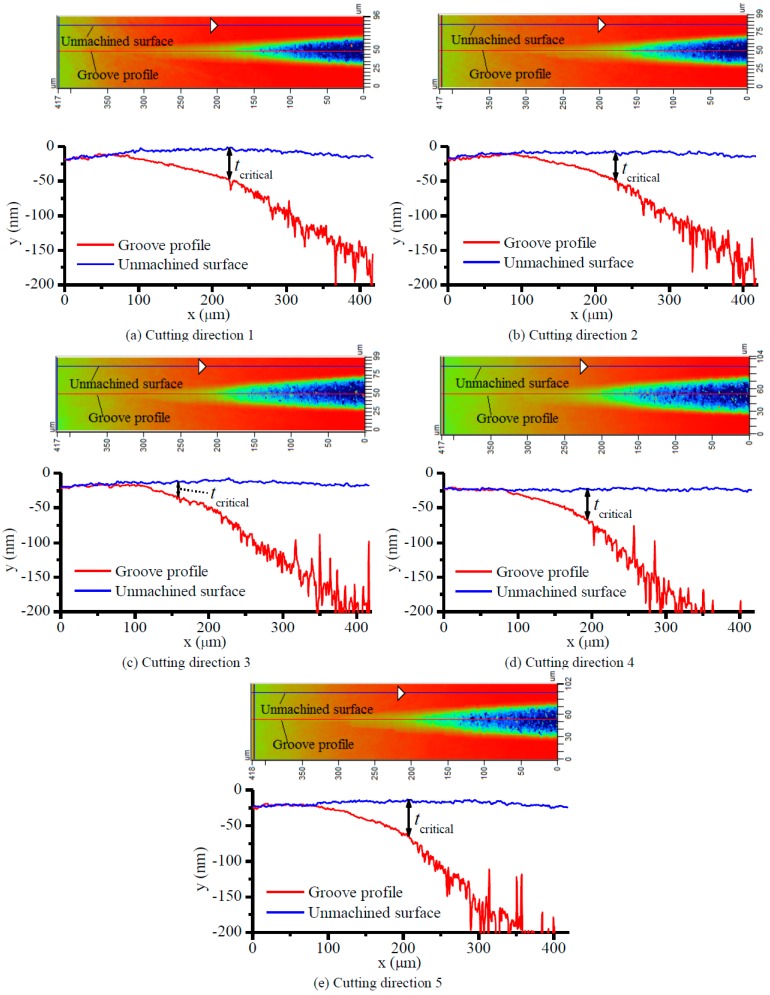
Profiles of the taper grooves.

Figure 12 shows the profiles of the taper grooves in the sections denoted as “unmachined surface” and “groove profile”. The sections denoted as “unmachined surface” were located on the unmachined surface and were parallel to the sections denoted as “groove profile”. Due to the circular form error of the diamond tool, the deepest sections of the grooves were not exactly located in the middle of the taper grooves. Therefore, the sections denoted as “groove profile” were adjusted to be located in the deepest sections in the grooves, and as a result might not seem to be positioned exactly in the middle of the taper grooves shown in Figure 12. Due to the flatness error of the fixture and the uneven thickness of the glue, the SiC sample might be slightly distorted after being glued onto the fixture. This might be the reason why the profiles of the unmachined surfaces appear to be slightly curved in Figure 12a,e. 

The profiles shown in Figure 12 agree with the surface morphologies shown in Figure 11 in that the profile of each machined groove could be divided into two parts, i.e., a smooth part corresponding to ductile-cut surface, and a coarse part corresponding to brittle-cut surface. The unmachined parts of the groove profiles almost coincided with the profiles of the sections denoted as “unmachined surface”, as can be seen in Figure 12a–e. The critical undeformed chip thickness $t_{critical}$ for each groove was measured as the distance between the profiles of the sections “unmachined surface” and “groove profile” at the point where obvious fluctuations start to appear on the groove profile, which is denoted by a double-headed arrow in each figure. 

**Figure 13:**
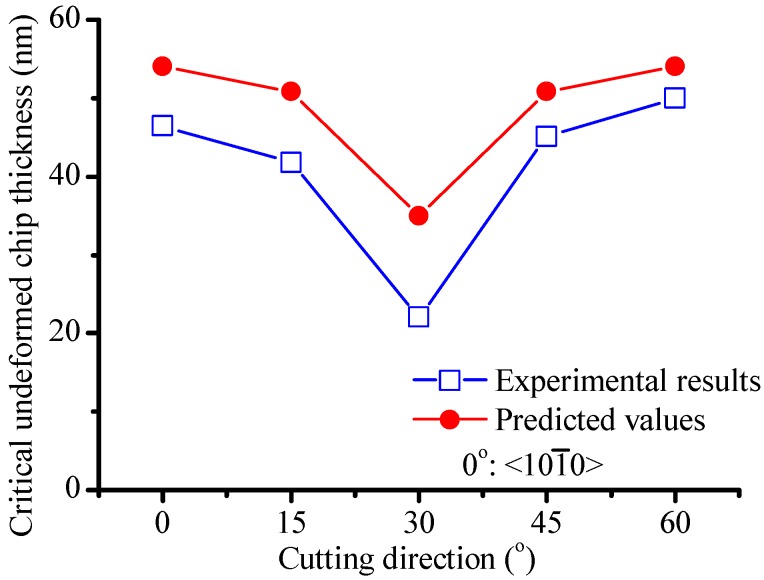
Predicted and experimental values of $t_{critical}$.

The experimentally measured critical undeformed chip thicknesses in the five cutting directions are plotted in Figure 13. The cutting directions are indicated by their angle to the <10$\overset{-}{1}$0> direction, which is the first cutting direction shown in Figure 10b. The experimental results varied between 22.1 and 50 nm in different crystal directions. For reference, Meng et al. [53] conducted nanoscratching of 6H-SiC and found that the depth of the groove at the transition zone was around 30 nm, but they did not mention the cutting direction. Jacob et al. [54] reported a $t_{critical}$ of 70 nm for 6H-SiC in fly cutting, also without mentioning the cutting direction. Since their tool rake angle was −40° which might increase the critical undeformed chip thickness [26,55], the experimental results presented in this study are reasonable, which were obtained under a tool rake angle of −30°. 

The critical undeformed chip thickness for cutting direction 1, 2 and 3 were estimated in Section 3.2. Due to crystal symmetry, the critical undeformed chip thicknesses for directions 4 and 5 should be the same with that for directions 2 and 1 respectively. The values of $t_{critical}$ predicted in this way are plotted in Figure 13 for comparison with the experimental results. It can be seen that the predicted variation of the critical undeformed chip thickness with cutting direction agrees very well with the experimental results. The predicted values generally are slightly higher than the experimental results. There are several possible reasons for the deviation. Firstly, the material properties calculated by molecular dynamics might be different with the practical sample. Secondly, as mentioned in Section 3.2, assuming the intersection of $\sigma_{xx}$ and $\sigma_{ct}$ in the cutting direction as the transition point might over-estimate the critical undeformed chip thickness. Thirdly, lattice defects might exist in the practical material, but were not taken into consideration in the modelling. Nevertheless, the predicted values agree quite well with the experimental results. The agreement between the predicted and experimental results of $t_{critical}$ again indicates the validity of the proposed mechanics of brittle–ductile cutting mode transition.

## 4. Discussion

The mechanism of brittle–ductile cutting mode transition was investigated from the perspective of mechanics in this study. The relationship between deformation, stress states, and undeformed chip thickness was mechanistically analyzed. The analysis of the mechanics in the cutting zone showed that, in order to reach a same level of maximum compressive stress, a larger undeformed chip thickness would require a larger distance of tool advancement and thereby a larger tensile stress would be induced below and behind the tool, which would cause brittle fracture when it exceeds the critical tensile stress for brittle fracture. The FEM modelling confirmed that, to reach a same magnitude of compressive stress, the tensile stress below and behind the tool would increase with the undeformed chip thickness, and would exceed the critical tensile stress for brittle fracture when the undeformed chip thickness increased to a certain level. Although the analysis of stress states is hard to be verified by experiments directly, the critical undeformed chip thickness estimated as the intersection of the increasing tensile stress and the critical tensile stress (see Figure 9) agreed well with the experimental results. In addition, the FEM modelling showed that the distance of tool advancement required to induce the same level of compressive stress increased with undeformed chip thickness (see Figure 8). This indicates that the proposed mechanics has its validity.

It needs to be mentioned that the aim of this study is to contribute a new insight into the mechanism of brittle–ductile cutting mode transition from another perspective, but not to replace the previous theories. In fact, the mechanics proposed in this study is consistent with previous theories in many aspects. Taking the theory from the perspective of energy [2] for example, the mechanics proposed in this study could be corresponded with this theory by referring to Figure 1. When the tool moves forward, compressive stress would be induced before the tool and tensile stress would be induced behind the tool. When the compressive stress exceeds the critical compressive stress for ductile flow or tensile stress exceeds the critical tensile stress for brittle fracture, ductile mode chip formation or brittle fracture would occur respectively. The developing of compressive and tensile stresses is associated with the accumulation of elastic energy, while the critical compressive and tensile stresses correspond to kind of critical elastic energies for ductile and brittle deformations. Therefore, the transition between the ductile and brittle modes of cutting could be viewed as the competition between the accumulated elastic energies. When the undeformed chip thickness is small, the accumulated elastic energy from compressive strains would exceed the critical elastic energy for ductile deformation before the accumulated elastic energy from tensile strains reaches the critical elastic energy for brittle fracture. When the undeformed chip thickness is large, the accumulated elastic energy from tensile strains would exceed the critical energy for brittle fracture before the accumulated elastic energy from compressive strains could induce ductile deformation. This is consistent with the theory from the energy perspective [2] that plastic flow would be energetically more favorable than brittle fracture when the deformation scale becomes small enough.

While the proposed mechanics is consistent with previous theories, it has its significance that it reveals an explicit relationship between undeformed chip thickness and stress states from the perspective of mechanics. Though previous studies have found that compressive stress would induce ductile chip formation under small undeformed chip thickness [16,17,18,19,20,21,22,23] and that tensile stress would cause brittle fracture under large undeformed chip thickness [22,34,35], the mechanics proposed in this study reveals an explicit relationship between the compressive and tensile stresses, and how their roles shift to determine the deformation mode when the undeformed chip thickness changes.

## 5. Conclusions

This study investigated the mechanism of brittle–ductile cutting mode transition from the perspective of mechanics in the cutting zone. The relationship between undeformed chip thickness and stress levels in the elastic stage of the periodic chip formation process was analyzed in terms of whether a ductile or brittle mode deformation stage is to follow the elastic stage. FEM modelling was conducted to verify the analysis of the mechanics, and to estimate the critical undeformed chip thickness according to the proposed mechanics. The estimated values of critical undeformed chip thickness agree well with the experimental results by plunge cutting. This study contributes a new insight into the mechanism of brittle–ductile cutting mode transition, by revealing an explicit relationship between undeformed chip thickness and the stress states from the perspective of mechanics. The following conclusions could be drawn from this study.

I. The distance of tool advancement required to induce the same magnitude of critical compressive stress decreases with undeformed chip thickness, and consequently the tensile stress induced below and behind the tool also decreases with undeformed chip thickness.
II. The tensile stress would become lower than the critical tensile stress for brittle fracture when the undeformed chip thickness becomes sufficiently small, and thereby becomes insufficient to induce brittle fractures. Hence the decrease of tensile stress with the decrease of undeformed chip thickness is considered to be the key factor for enabling brittle–ductile cutting mode transition.

## Figures and Tables

**Table 1:** Molecular dynamics (MD) simulation parameters for tension and compression.

Parameter	Value
Workpiece dimension	Φ8.7 nm × 17.4 nm
Total atoms	Around 101,600
Loading rate	1.74 m/s

**Table 2:** MD results of critical tensile and compressive stresses.

Orientation	$\sigma_{ct}$	$\sigma_{cc}$
(0001) <10$\overset{-}{1}$0>	24, 380 MPa	33, 744 MPa
(0001) <15 $\overset{-}{4}$ $\overset{-}{11}$ 0>	23, 364 MPa	35, 574 MPa
(0001) <2$\overset{-}{1}$$\overset{-}{1}$0>	22, 338 MPa	44, 718 MPa

**Table 3:** Cutting parameters adopted in the FEM modelling.

Parameter	Value
Tool rake angle	−30°
Clearance angle	10°
Edge radius (nm)	50
$t_{undefomed}$ (nm)	30, 35, 40, 45, 50, 55, 60
Cutting speed (mm/s)	3
Cutting plane	(0001)
Cutting direction	<10$\overset{-}{1}$0>, <15 $\overset{-}{4}$ $\overset{-}{11}$ 0>, <2$\overset{-}{1}$$\overset{-}{1}$0>

**Table 4:** Cutting parameters for the plunge cutting experiments.

Parameter	Value
Tilt angle	~0.03°
Rake angle	−30°
Clearance angle	10°
Cutting speed	3 mm/s

## References

[1] Blackley W.S., Scattergood R.O. (1991). Ductile-regime machining model for diamond turning of brittle materials. Precis. Eng..

[2] Bifano T.G., Dow T.A., Scattergood R.O. (1991). Ductile-regime grinding: A new technology for machining brittle materials. J. Eng. Ind..

[3] Arif M., Xinquan Z., Rahman M., Kumar S. (2013). A predictive model of the critical undeformed chip thickness for ductile-brittle transition in nano-machining of brittle materials. Int. J. Mach. Tools Manuf..

[4] Cheng J., Wu J., Gong Y.D., Wen X.L., Wen Q. (2016). Experimental study on the single grit interaction behaviour and brittle–ductile transition of grinding with a diamond micro-grinding tool. Int. J. Adv. Manuf. Technol..

[5] Mizumoto Y., Maas P., Kakinuma Y., Min S. (2017). Investigation of the cutting mechanisms and the anisotropic ductility of monocrystalline sapphire. CIRP Ann.-Manuf. Technol..

[6] Yang M., Li C., Zhang Y., Jia D., Zhang X., Hou Y., Li R., Wang J. (2017). Maximum undeformed equivalent chip thickness for ductile-brittle transition of zirconia ceramics under different lubrication conditions. Int. J. Mach. Tools Manuf..

[7] Chen X., Xu J., Fang H., Tian R. (2017). Influence of cutting parameters on the ductile-brittle transition of single-crystal calcium fluoride during ultra-precision cutting. Int. J. Adv. Manuf. Technol..

[8] Chen J.B., Fang Q.H., Wang C.C., Du J.K., Liu F. (2016). Theoretical study on brittle–ductile transition behavior in elliptical ultrasonic assisted grinding of hard brittle materials. Precis. Eng..

[9] Zhou M., Zhao P. (2016). Prediction of critical cutting depth for ductile-brittle transition in ultrasonic vibration assisted grinding of optical glasses. Int. J. Adv. Manuf. Technol..

[10] Nakasuji T., Kodera S., Hara S., Matsunaga H., Ikawa N., Shimada S. (1990). Diamond turning of brittle materials for optical components. CIRP Ann.-Manuf. Technol..

[11] Shimada S., Ikawa N., Inamura T., Takezawa N., Ohmori H., Sata T. (1995). Brittle–ductile transition phenomena in microindentation and micromachining. CIRP Ann.-Manuf. Technol..

[12] Ueda K., Sugita T., Tsuwa H. (1983). Application of fracture mechanics in micro-cutting of engineering ceramics. CIRP Ann.-Manuf. Technol..

[13] Ueda K., Sugita T., Hiraga H., Iwata K. (1991). A j-integral approach to material removal mechanisms in microcutting of ceramics. CIRP Ann.-Manuf. Technol..

[14] Hiatt G.D. (1992). A Fracture Mechanics Technique for Predicting the Ductile Regime in Single Point Diamond Turning of Brittle Materials. Ph.D. Thesis.

[15] Venkatachalam S., Li X., Liang S.Y. (2009). Predictive modeling of transition undeformed chip thickness in ductile-regime micro-machining of single crystal brittle materials. J. Mater. Process. Technol..

[16] Fang F., Venkatesh V. (1998). Diamond cutting of silicon with nanometric finish. CIRP Ann.-Manuf. Technol..

[17] Liu K., Li X. (2001). Ductile cutting of tungsten carbide. J. Mater. Process. Technol..

[18] Yan J., Yoshino M., Kuriagawa T., Shirakashi T., Syoji K., Komanduri R. (2001). On the ductile machining of silicon for micro electro-mechanical systems (MEMS), opto-electronic and optical applications. Mater. Sci. Eng. A.

[19] Yan J., Syoji K., Tamaki J.I. (2004). Crystallographic effects in micro/nanomachining of single-crystal calcium fluoride. J. Vac. Sci. Technol. B.

[20] Liu K., Li X., Rahman M., Neo K., Liu X. (2007). A study of the effect of tool cutting edge radius on ductile cutting of silicon wafers. Int. J. Adv. Manuf. Technol..

[21] Liu K., Li X., Liang S. (2007). The mechanism of ductile chip formation in cutting of brittle materials. Int. J. Adv. Manuf. Technol..

[22] Yan J., Syoji K., Kuriyagawa T., Suzuki H. (2002). Ductile regime turning at large tool feed. J. Mater. Process. Technol..

[23] Fang F., Wu H., Zhou W., Hu X. (2007). A study on mechanism of nano-cutting single crystal silicon. J. Mater. Process. Technol..

[24] Morris J., Callahan D. (1994). Origins of microplasticity in low-load scratching of silicon. J. Mater. Res..

[25] Tanikella B., Somasekhar A., Sowers A., Nemanich R., Scattergood R. (1996). Phase transformations during microcutting tests on silicon. Appl. Phys. Lett..

[26] Patten J.A., Gao W., Yasuto K. (2005). Ductile regime nanomachining of single-crystal silicon carbide. J. Manuf. Sci. Eng..

[27] Tanaka H., Shimada S., Anthony L. (2007). Requirements for ductile-mode machining based on deformation analysis of mono-crystalline silicon by molecular dynamics simulation. CIRP Ann.-Manuf. Technol..

[28] Ezugwu E.O., Bonney J. (2004). Effect of high-pressure coolant supply when machining nickel-base, inconel 718, alloy with coated carbide tools. J. Mater. Process. Technol..

[29] Maruda R.W., Krolczyk G.M., Nieslony P., Wojciechowski S., Michalski M., Legutko S. (2016). The influence of the cooling conditions on the cutting tool wear and the chip formation mechanism. J. Manuf. Process..

[30] Maruda R.W., Legutko S., Krolczyk G.M., Raos P. (2015). Influence of cooling conditions on the machining process under MQCL and MQL conditions. Teh. Vjesn.-Tech. Gaz..

[31] Vagnorius Z., Sorby K. (2011). Effect of high-pressure cooling on life of SiAlON tools in machining of Inconel 718. Int. J. Adv. Manuf. Technol..

[32] Cai M., Li X., Rahman M. (2007). Study of the mechanism of nanoscale ductile mode cutting of silicon using molecular dynamics simulation. Int. J. Mach. Tools Manuf..

[33] Xiao G., To S., Zhang G. (2015). Molecular dynamics modelling of brittle–ductile cutting mode transition: Case study on silicon carbide. Int. J. Mach. Tools Manuf..

[34] Patten J.A. Cutting tool edge radius and depth of cut influence on the generation of the high pressure phase transformation during ductile machining. Proceedings of the ASPE Spring Topical Meeting on Silicon Machining.

[35] Blackley W., Scattergood R.O. (1994). Chip topography for ductile-regime machining of germanium. J. Manuf. Sci. Eng..

[36] Astakhov V.P., Osman M.O.M., Hayajneh M.T. (2001). Re-evaluation of the basic mechanics of orthogonal metal cutting: Velocity diagram, virtual work equation and upper-bound theorem. Int. J. Mach. Tools Manuf..

[37] Inamura T., Takezawa N., Kumaki Y., Sata T. (1994). On a possible mechanism of shear deformation in nanoscale cutting. CIRP Ann.-Manuf. Technol..

[38] Wang H., Riemer O., Rickens K., Brinksmeier E. (2016). On the mechanism of asymmetric ductile-brittle transition in microcutting of (111) CaF_2_ single crystals. Scr. Mater..

[39] Yan J., Zhao H., Kuriyagawa T. (2009). Effects of tool edge radius on ductile machining of silicon: An investigation by fem. Semicond. Sci. Technol..

[40] Wang S., An C., Zhang F., Wang J., Lei X., Zhang J. (2016). An experimental and theoretical investigation on the brittle ductile transition and cutting force anisotropy in cutting KDP crystal. Int. J. Mach. Tools Manuf..

[41] Shibata T., Fujii S., Makino E., Ikeda M. (1996). Ductile-regime turning mechanism of single-crystal silicon. Precis. Eng..

[42] Leung T.P., Lee W.B., Lu X.M. (1998). Diamond turning of silicon substrates in ductile-regime. J. Mater. Process. Technol..

[43] O’Connor B.P., Marsh E.R., Couey J.A. (2005). On the effect of crystallographic orientation on ductile material removal in silicon. Precis. Eng..

[44] Chen H., Dai Y., Zheng Z., Gao H., Li X. (2011). Effect of crystallographic orientation on cutting forces and surface finish in ductile cutting of KDP crystals. Mach. Sci. Technol..

[45] Dornfeld D.A., Lee D.E. (2008). Precision Manufacturing.

[46] Adachi S. (2004). Handbook on Physical Properties of Semiconductors.

[47] Vashishta P., Kalia R.K., Nakano A., Rino J.P. (2007). Interaction potential for silicon carbide: A molecular dynamics study of elastic constants and vibrational density of states for crystalline and amorphous silicon carbide. J. Appl. Phys..

[48] Kikuchi H., Kalia R.K., Nakano A., Vashishta P., Branicio P.S., Shimojo F. (2005). Brittle dynamic fracture of crystalline cubic silicon carbide (3c-sic) via molecular dynamics simulation. J. Appl. Phys..

[49] Coati A., Sauvage-Simkin M., Garreau Y., Pinchaux R., Argunova T., Aid K. (1999). ($\sqrt{3}\times \sqrt{3}$) *R*30° reconstruction of the 6H-SiC (0001) surface: A simple T4 Si adatom structure solved by grazing-incidence X-ray diffraction. Phys. Rev. B.

[50] Stukowski A. (2010). Visualization and analysis of atomistic simulation data with ovito—The open visualization tool. Model. Simul. Mater. Sci. Eng..

[51] Gao W. (2010). Precision Nanometrology: Sensors and Measuring Systems for Nanomanufacturing.

[52] Yang W., Liu X., Lu W., Yu N., Chen L., Zhou L., Chang S. (2017). A novel white light interference based AFM head. J. Lightw. Technol..

[53] Meng B., Zhang F., Li Z. (2015). Deformation and removal characteristics in nanoscratching of 6H-SiC with berkovich indenter. Mater. Sci. Semicond. Process..

[54] Jacob J., Patten J.A., Bhattacharya B., Couey J.A., Marsh E.R. Determination of the ductile to brittle transition and critical depth of cut in 6H-silicon carbide through fly cutting. Proceedings of the ASPE Annual Meeting.

[55] Chiu W.C., Endres W.J., Thouless M. (2000). An experimental study of orthogonal machining of glass. Mach. Sci. Technol..

